# Papers on Cholera

**Published:** 1867-06

**Authors:** 


### PAPERS ON CHOLERA.

The Secretary presented a paper upon the Cause and Management of Cholera, by Dr. E. Harris, which was referred to a committee to examine papers designed for publication.

Dr. Wm. Marsden, of Canada, presented a synopsis of an essay on the contagion, infection, and portability of Asiatic cholera, in its relations to quarantine, with a brief history of its origin and cause in Canada, from 1832.

A motion to refer to the Committee on Publication was adopted, but afterward reconsidered, and the paper referred to the Committee on Revision, above authorized.

